# Galanin System in Human Glioma and Pituitary Adenoma

**DOI:** 10.3389/fendo.2020.00155

**Published:** 2020-03-24

**Authors:** Sarah Falkenstetter, Julia Leitner, Susanne M. Brunner, Tim N. Rieder, Barbara Kofler, Serge Weis

**Affiliations:** ^1^Research Program for Receptor Biochemistry and Tumor Metabolism, Department of Pediatrics, University Hospital of the Paracelsus Medical University, Salzburg, Austria; ^2^Division of Neuropathology, Department of Pathology and Neuropathology, Neuromed, School of Medicine Campus, Kepler University Hospital, Johannes Kepler University, Linz, Austria

**Keywords:** galanin receptor, neuropeptide, brain tumor, glioma, pituitary adenoma, macrophage

## Abstract

Expression of neuropeptides and their corresponding receptors has been demonstrated in different cancer types, where they can play a role in tumor cell growth, invasion, and migration. Human galanin (GAL) is a 30-amino-acid regulatory neuropeptide which acts through three G protein-coupled receptors, GAL_1_-R, GAL_2_-R, and GAL_3_-R that differ in their signal transduction pathways. GAL and galanin receptors (GALRs) are expressed by different tumors, and direct involvement of GAL in tumorigenesis has been shown. Despite its strong expression in the central nervous system (CNS), the role of GAL in CNS tumors has not been extensively studied. To date, GAL peptide expression, GAL receptor binding and mRNA expression have been reported in glioma, meningioma, and pituitary adenoma. However, data on the cellular distribution of GALRs are sparse. The aim of the present study was to examine the expression of GAL and GALRs in different brain tumors by immunohistochemistry. Anterior pituitary gland (*n* = 7), pituitary adenoma (*n* = 9) and glioma of different WHO grades I–IV (*n* = 55) were analyzed for the expression of GAL and the three GALRs with antibodies recently extensively validated for specificity. While high focal GAL immunoreactivity was detected in up to 40% of cells in the anterior pituitary gland samples, only one pituitary adenoma showed focal GAL expression, at a low level. In the anterior pituitary, GAL_1_-R and GAL_3_-R protein expression was observed in up to 15% of cells, whereas receptor expression was not detected in pituitary adenoma. In glioma, diffuse and focal GAL staining was noticed in the majority of cases. GAL_1_-R was observed in eight out of nine glioma subtypes. GAL_2_-R immunoreactivity was not detected in glioma and pituitary adenoma, while GAL_3_-R expression was significantly associated to high-grade glioma (WHO grade IV). Most interestingly, expression of GAL and GALRs was observed in tumor-infiltrating immune cells, including neutrophils and glioma-associated macrophages/microglia. The presence of GALRs on tumor-associated immune cells, especially macrophages, indicates that GAL signaling contributes to homeostasis of the tumor microenvironment. Thus, our data indicate that GAL signaling in tumor-supportive myeloid cells could be a novel therapeutic target.

## Introduction

Malignant brain tumors are the most common cause of cancer-related deaths in adolescents and young adults aged 15–39 and the most common cancer occurring among 15–19 year olds (1). Due to the diffuse infiltration into the brain by various brain cancer types, such as glioma (i.e., astrocytoma and oligodendroglioma; WHO grade I–IV), surgical intervention is difficult and often limited (2). Consequently, there is an urgent need to understand tumor biology and subsequently identify new drug targets for the treatment of brain tumors.

Possible new drug candidates might be found in the group of neuropeptides. Neuropeptide expression has been shown in many different cancer types, and neuropeptide expression levels correlate with tumor differentiation or aggressive behavior. Thus, neuropeptides could be useful for tumor imaging and as biomarkers for prognosis. More importantly, neuropeptides are involved in tumor cell growth, invasion, and migration (3–5), supporting their potential in developing novel anti-tumor treatment strategies.

Human galanin (GAL) is a 30-aa regulatory neuropeptide which plays a role in several physiological processes. Its functions are mediated by the G protein-coupled receptors GAL_1_-R, GAL_2_-R, and GAL_3_-R that differ in their signal transduction pathways. GAL_1_-R and GAL_3_-R predominantly couple to Gi/o, leading to a reduction of cAMP and consequently an inactivation of the protein kinase A (PKA). GAL_2_-R signals via multiple classes of G proteins, but preferably via Gq/11, which results in the activation of the protein kinase C (PKC). GALRs show sequence homologies, particularly in the transmembrane regions. GAL_1_-R and GAL_3_-R show 33% sequence homology, whereas GAL_2_-R and GAL_3_-R show 54% sequence homology (6). Besides species-specific expression patterns of GAL and galanin receptors (GALRs), expression is also tissue-specific. GAL is expressed in neuronal and endocrinal tissues at highest levels. In addition, GALRs are expressed in different tissues, with GAL_1_-R mRNA in particular being strongly expressed in the brain. GAL_2_-R mRNA is less abundant and restricted to certain brain regions, whereas GAL_3_-R mRNA is more restricted to peripheral tissues (6). Recently, expression of GAL and GALRs in human immune cells such as neutrophils and macrophages was also reported (7).

Human pheochromocytoma was the first tumor in which GAL was identified (8, 9). Later, GAL-like immunoreactivity was detected in other neuroendocrine tumors, including human pituitary adenoma, particularly associated with adrenocorticotrophic hormone-secreting cells (10–16), and gangliocytoma (14, 17), paraganglioma (18, 19), and neuroblastoma (20). GAL has also been detected in a variety of non-neuroendocrine human tumors of different origin, including glioblastoma and other brain tumors (21), melanoma (22), head and neck squamous cell carcinoma (HNSCC) (23), basal cell carcinoma (24), colon cancer (25–27) and embryonic carcinoma (28). Interestingly, the majority of these tumors exhibited significantly higher GAL levels than corresponding non-cancerous tissue (22, 23, 25, 27, 28). In colon cancers, GAL mRNA levels correlated with tumor size and stage (25), for which a significant correlation between high GAL expression and shorter disease-free survival in colon cancer patients was observed (27).

In humans, GALRs were first discovered in pituitary tumors (29) and subsequently identified in pheochromocytoma (30), neuroblastoma (20), glioma (21), prostate carcinoma (30), colon carcinoma (27), and HNSCC (31). GAL_1_-R mRNA is the most abundantly expressed GALR mRNA in human meningioma, glioblastoma (21) and neuroblastoma (32). Elevated GAL_1_-R mRNA expression is associated with increased malignancy (33). Increased GAL_1_-R mRNA expression was also observed in human pituitary adenoma relative to levels in normal human pituitary gland (34), suggesting cancer-promoting properties for GAL_1_-R at least in these tumors. Furthermore, activation of GAL_1_-R induces cell-cycle arrest and suppresses proliferation of HNSCC cell lines (31, 35, 36). Anti-proliferative effects via GAL_1_-R signaling have also been observed in human SH-SY5Y neuroblastoma cells transfected with GAL_1_-R (37).

In contrast, the presence of GAL_2_-R mRNA is less common in human glioma (21) and neuroblastoma (20). GAL_2_-R mRNA expression is low in the majority of human pituitary adenomas compared to levels in normal human pituitary (34). However, elevated GAL_2_-R mRNA expression was observed in human pheochromocytoma (38).

It is noteworthy that transfection of GAL_2_-R into human SH-SY5Y neuroblastoma cells and into human HNSCC cells led to suppressed cell proliferation and induction of caspase-dependent apoptosis (36–40). On the other hand, in small cell lung cancer, activation of GAL_2_-R exerted growth-promoting effects (41, 42).

The impact of GAL_3_-R signaling on the biological activity of cancer cells is less well-studied. GAL_3_-R expression was detected in neuroblastoma (32, 33) and glioma (21). Analysis of human HNSCC revealed significantly increased GAL_3_-R expression in the tumors compared to normal tissue (23). Similarly, GAL_3_-R mRNA expression was detected in human pituitary adenoma associated with tumor relapse, whereas it was absent in post-mortem pituitary glands (34).

To date, GAL-binding studies have been used to deduce the presence of GALRs in human glioma, meningioma (21) and pituitary adenoma (29), but no receptor subtype has been identified at the cellular level, except indirectly from mRNA expression analyses in tissue extracts (21, 34). Thus, information on the cellular distribution of GALRs has been missing due to a lack of specific GAL receptor antibodies. Recently, we were able to identify specific anti-human GALR- specific antibodies, which now allow us to determine the distribution of the three GALRs at the cellular level (43).

The aim of the present study was to elucidate the expression of GAL and GALRs in different human brain tumors by immunohistochemistry (IHC) with carefully validated antibodies.

## Materials and Methods

### Ethics Statement

Experiments were conducted in accordance with the Helsinki Declaration of 1975 (revised 1983) and the guidelines of the Salzburg State Ethics Research Committee (AZ2 09-11-E1/823-2006), being no clinical drug trial or epidemiological investigation. In accordance with the Upper Austrian Ethics Committee, upon hospital admission, patients signed an informed consent document concerning the surgical intervention, and agreed to the use of the surgically removed tumor tissue for research purposes. Furthermore, the study did not extend to examination of individual case records. Patient anonymity was ensured at all times. Cancer tissues were derived from surgery. Pituitary glands were obtained post mortem from patients with no signs of brain tumors who died due to either cardiorespiratory failure or brain hemorrhage. Demographics of individual patients are provided in Supplementary Tables 1–3.

### Patients and Material

Formalin-fixed paraffin-embedded (FFPE) tumor tissue of glioma and pituitary adenoma as well as anterior pituitary glands were provided by the Division of Neuropathology, Neuromed Campus, Kepler University Hospital, Linz, Austria.

In total, 55 glioma and 9 pituitary adenoma samples were analyzed for the expression of GAL and GALRs by IHC. Detailed information on tumor subtypes and WHO grades, including astrocytic tumors (*n* = 37), oligodendroglial tumors (*n* = 15) and mixed neuronal-glial tumors (*n* = 3), and age of the patients is provided in Table 1 and Supplementary Tables 2, 3. Data on 7 anterior pituitary glands used for antibody validation are also included (Supplementary Table 1). The neuropathology diagnosis was based on the diagnostic criteria outlined in the revised 4th edition of the WHO Classification of tumors of the CNS (44). Briefly, the mutation status of the *IDH1* and *IDH2* genes was assessed for astroglioma and oligodendroglioma; 1p19q co-deletion was determined for oligodendroglioma using multiplex ligation-dependent probe amplification. Cases with a former diagnosis of oligoastrocytoma were re-evaluated using the above-mentioned molecular diagnostic parameters. Two cases of oligoastrocytoma could not be assigned to astroglioma or oligodendroglioma and are therefore described separately. Their data are not included in the statistics.

**Table 1:** Information on tumor samples (incl. WHO classification and grade, sample size (n), and patient age range), positive-stained samples (%), as well as the range of positive-stained cells in (%) and the range of staining intensity.

**Tumor type**	**WHO grade**	***n***	**Median age [age range] (years)**	**Diffuse GAL staining**	**Focal GAL staining**	**GAL_**1**_-R**	**GAL_**2**_-R**	**GAL_**3**_-R**
				**positive-stained samples (%) range of positive-stained cells (%), intensity range (0–3)**				
Pilocytic astrocytoma	I	5	16 [3–20]	100% 0–2	60% <1–18%, 1–3	20% <1%, 1	0%	20% <1%, 2–3
Diffuse astrocytoma	II	7	39 [4–76]	100% 0–2	57% 2–40%, 1–3	0%	0%	0%
Anaplastic astrocytoma	III	7	33 [4–61]	100% 0–2	86% <1–65%, 1–2	43% <1%, 1	0%	0%
Glioblastoma multiforme	IV	8	62 [21–75]	100% 0–2	75% <1–30%, 1–2	38% <1–8%, 1–2	0%	63% <1–7%, 1–3
Gliosarcoma	IV	6	55 [40–68]	100% 0–2	50% 15–70%, 1–2	33% <1–1%, 1	0%	50% <1–2%, 1–2
Giant cell glioblastoma	IV	4	42 [24–76]	100% 0–1	100% 35–80%, 1–2	50% <1%, 1–2	0%	0%
Oligodendroglioma	II	9	38 [20–76]	89% 0–2	78% <1–10%, 1–2	22% <1%, 1–2	0%	11% <1%, 1–2
Anaplastic oligodendroglioma	III	6	36 [31–37]	67% 0–2	67% <1–30%, 1–3	17% 1%, 1–2	0%	17% <1%, 1–2
Ganglioglioma	I	3	21 [4–21]	67% 0–2	67% <1–6%, 2–3	67% <1%, 1	0%	67% <1–3%, 1–2
Pituitary adenoma		9	57 [27–74]	89% 0–2	11% 2%, 2–3	0%	0%	0%
Anterior pituitary gland		7	78 [61–92]	100% 1–2	100% 2–40%, 3	100% 7–15%, 3	0%	100% <1–5%, 2–3

### Immunohistochemistry

For IHC analysis, 4 μm FFPE tissue sections were stained as described previously (45) using the Envision+ System-HRP (DAB) Kit (DAKO, Glostrup, Denmark). After drying for 1 h at 60°C, sections were deparaffinized and rehydrated. Epitope retrieval was performed with EDTA-Tris buffer (1 mM EDTA, 10 mM Tris, pH 9) for 40 min at 95°C. After blocking endogenous peroxidases with “Peroxidase blocking solution” (DAKO), the primary antibody diluted in “Antibody Diluent with Background Reducing Components” (DAKO) was added (40 min, 37°C). The following polyclonal antibodies were used: anti-GAL (Peninsula/Bachem, San Carlos, CA, USA, T-4325, LOT: A14907, rabbit, 1:300), anti-GAL_1_-R (GeneTex Inc., Irvine, CA, USA, GTX108207, LOT: 39771, rabbit, 1:200), anti-GAL_2_-R [Proteintech Group Inc., Rosemont, IL, USA, customized, LOT: S4510-1, rabbit, 1:400; (45)] and anti- GAL_3_-R (GeneTex Inc., Irvine, CA, USA, GTX108163, LOT: 39764, rabbit, 1:500). The specificity of the antibodies against human GALRs was recently demonstrated (43, 45). Subsequently, the anti-rabbit secondary antibody “Envision+HRP-labeled polymer” (DAKO) was added for 30 min at RT. For visualization, “Envision+Liquid DAB+Chromogen” (DAKO) was applied (10 min, RT). Mayer's hemalum solution (Merck KGaA, Darmstadt, Germany) was used for counterstaining (3–5 min). Slides were immersed in 0.75% HCl in ethanol and rinsed under running tap water (10 min). After dehydration, the slides were mounted with Histokitt (Karl Hecht GmbH & Co KG, Sondheim, Germany). Digital micrographs were taken with a Moticam 5+ camera using Motic Image Plus 2.0 software (Motic, Wetzlar, Germany).

For each round of IHC staining, appropriate control sections were included as quality control. Human skin sections were used as positive controls for GAL [epidermis, sweat glands (46, 47)] and GAL_3_-R [blood vessels (48)]. The cell line SH-SY5Y transfected with human GAL_1_-R or human GAL_2_-R was used as a positive control for GAL_1_-R and GAL_2_-R staining [Supplementary Figures 1, 2; (43, 45)]. Furthermore, as a control, the primary antibody was omitted.

The percentage of stained tumor cells was estimated, excluding adjacent normal appearing tissue, as well as necrotic or hemorrhagic areas. If a section contained <10 positive-stained cells, the staining was regarded as negative. The staining intensity of tumor cells was rated from negative (0) to strong (3). IHC staining of non-tumor cells, vessels and immune cells such as neutrophils and glioma-associated macrophages/microglia (GAMs) was also evaluated. The IHC analysis was performed by two independent observers.

### RT-PCR Analysis

RNA was isolated from frozen tissue with Tri Reagent (Molecular Research Center Inc., Cincinnati, OH, USA) according to the manufacturer's instructions. Two micrograms of human RNA were used to generate cDNA by using maxima reverse transcriptase (Thermo Fisher, Waltham, MA, USA) following the manufacturer's protocol. Expression levels were quantified via qPCR using SYBR green SuperMix (BioRad, Hercules, CA, USA). The amplification was performed for 40 cycles (97°C for 15 s, 63°C for 30 s, and 72°C for 10 s) with specific primers for the genes of interest (Supplementary Table 4).

Relative expression levels of all genes were calculated as differences between the threshold cycle (Ct) of the gene of interest and Ct of the human housekeeping gene ribosomal protein L27 (RPL27).

### Statistical Analysis

Cramer-V was used to compare the expression of GAL and GALRs among 55 glioma cases with astrocytic, oligodendroglial, and mixed neuronal-glial tumor subclasses as appropriate. Cramer-V and Fisher's exact test were used to compare the expression of GAL and GALRs in glioma of different WHO grades. *P* < 0.05 was considered significant. All analyses were performed using SPSS 24.0 (SPSS Inc., Chicago, IL., USA).

## Results

### Antibody Validation on Human Anterior Pituitary Glands

Although the antibodies used in the present studies had been validated on peripheral tissues and overexpressing cell lines, we first validated the IHC protocol for healthy brain tissue, processed the same way as the tumor tissues, and compared the results to mRNA expression data. From seven healthy anterior pituitary glands, half the tissue was fixed in formaldehyde and processed for IHC analysis whereas the other half of the gland was fresh frozen for subsequent mRNA expression analysis.

**Figure 1:**
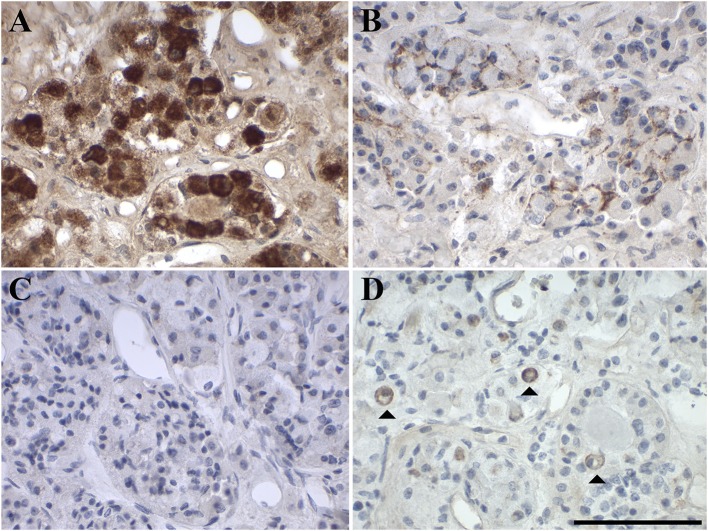
Representative images of immunohistochemical staining of **(A)** GAL, **(B)** GAL_1_-R, **(C)** GAL_2_-R, and **(D)** GAL_3_-R in human anterior pituitary gland (case 1). [scale bar: 100 μm].

**Figure 2:**
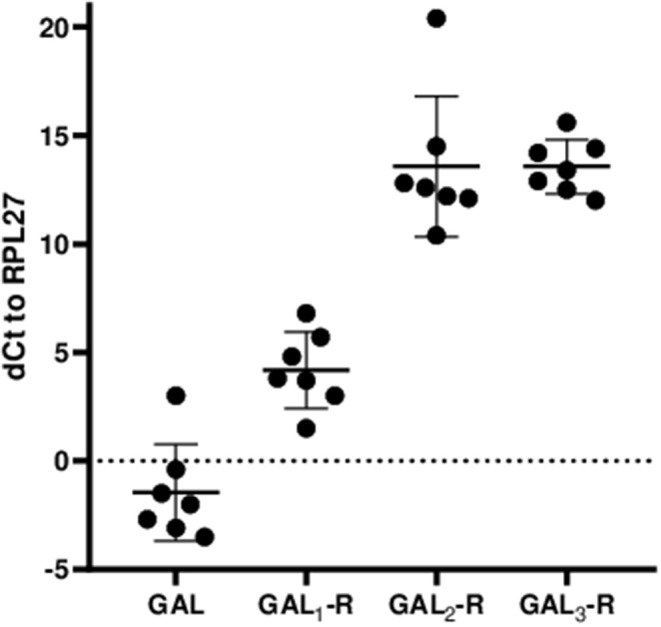
Relative mRNA expression levels of GAL and GALRs in anterior pituitary glands are shown as ΔCt values relative to the human housekeeping gene hRPL27. The values represent mean ± SD (*n* = 7).

IHC analysis revealed very strong focal intracellular GAL-immunoreactivity (2–40% of cells) in the pituitary glands. The remaining cells showed diffuse staining for GAL. This diffuse staining might be due to low expression levels and also to intercellular GAL secreted by high- GAL-expressing cells (Figure 1A). The high expression levels of the GAL peptide were also confirmed by RT-PCR analysis (Figure 2).

GAL_1_-R was the most prominent receptor in the anterior pituitary gland, with 7–15% positive membrane-associated cellular staining (medium to high staining intensity). GAL_2_-R-immunoreactivity was not detectable in anterior pituitary gland, whereas <1–5% of cells showed membrane-associated GAL_3_-R-immunoreactivity (medium to high staining intensity; Figure 1, Table 1, Supplementary Table 1).

The IHC staining results correlate well with mRNA expression, with GAL_1_-R being the most prominent galanin receptor at the mRNA level, followed by GAL_2_-R and GAL_3_-R, which on average showed the same ΔCt values (Figure 2).

#### Expression of GAL and GALRs in Pituitary Adenoma

**Figure 3:**
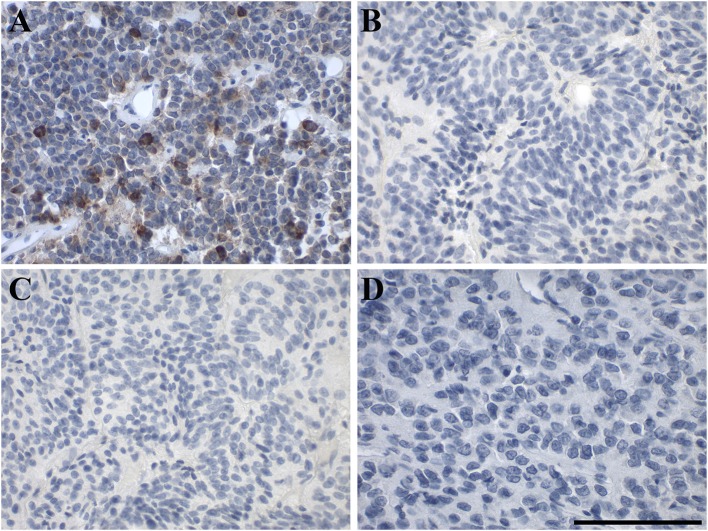
Representative images of immunohistochemical staining of in human pituitary adenoma. **(A)** GAL (case 7), **(B)** GAL_1_-R (case 5), **(C)** GAL_2_-R (case 5), and **(D)** GAL_3_-R (case 8). [scale bar: 100 μm].

Out of 9 pituitary adenomas, only one, a null-cell tumor (case 7), was positive for GAL, showing medium to strong focal GAL-immunoreactivity in 2% of the tumor cells (Figure 3A). Three pituitary adenomas [cases 3 (FSH), 5 (null cell), 6 (STH, prolactin)] had <10 GAL-positive stained cells and the remaining five were negative for focal GAL staining. Eight pituitary adenomas displayed diffuse GAL-immunoreactivity with low to medium intensity. Case 1 (prolactin) remained completely negative for diffuse and focal GAL staining. None of the GALRs was detectable in pituitary adenoma by IHC staining (Figures 3B–D, Table 1, Supplementary Table 2).

#### Expression of GAL and GALRs in Gliomas

**Figure 4:**
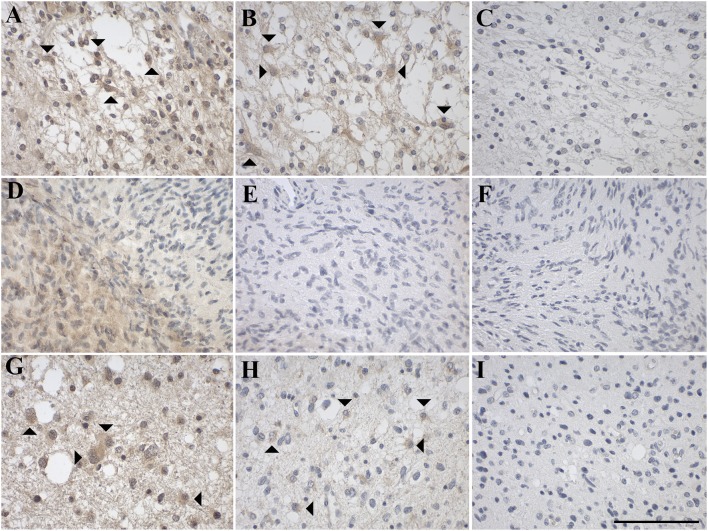
Representative images of immunohistochemical staining of low-grade astrocytic tumors with **(A–C)** pilocytic astrocytoma (WHO grade I; case 1), **(D–F)** diffuse astrocytoma (WHO grade II; case 8), and **(G–I)** anaplastic astrocytoma (WHO grade III; case 12). GAL immunoreactivity is shown in **(A,D,G)**; GAL_1_-R immunoreactivity in **(B,E,H)**, and GAL_3_-R immunoreactivity in **(C,F,I)**. Arrow heads indicate positive-stained glial cells. [scale bar: 100 μm].

**Figure 5:**
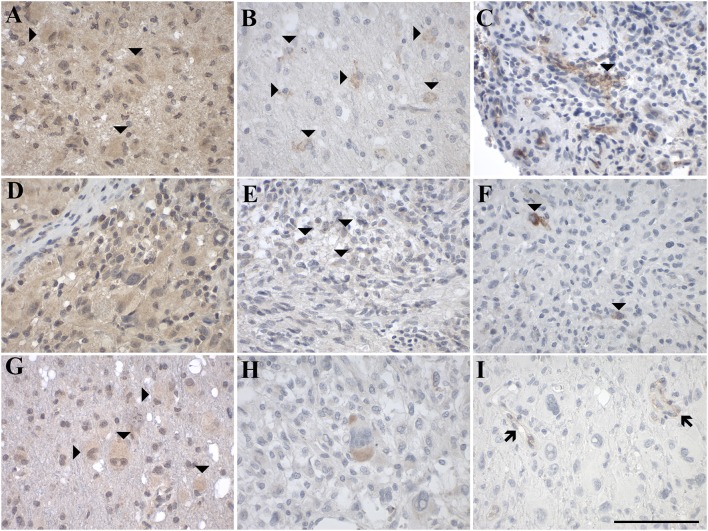
Representative images of immunohistochemical staining of high-grade astrocytic tumors with **(A–C)** glioblastoma multiforme (WHO grade IV; case 18, 17, 20), **(D–F)** gliosarcoma (WHO grade IV; case 27, 25, 27), and **(G–I)** giant cell glioblastoma (WHO grade IV; case 31, 30, 31). GAL immunoreactivity is shown in **(A,D,G)**; GAL_1_-R immunoreactivity in **(B,E,H)**, and GAL_3_-R immunoreactivity in **(C,F,I)**. Arrow heads indicate positive-stained cells whereas the arrows point out GAL_3_-R-positive blood vessels. [scale bar: 100 μm].

**Figure 6:**
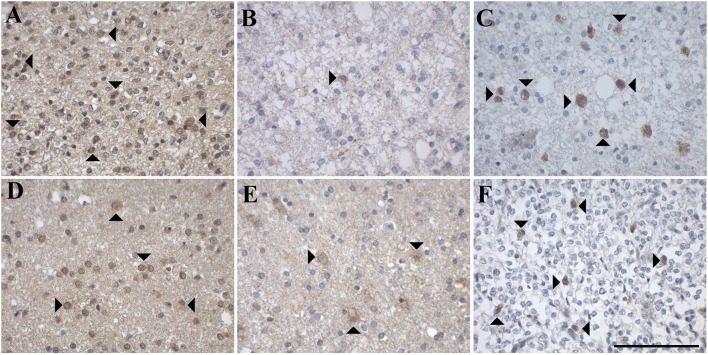
Representative images of immunohistochemical stainings of oligodendroglial tumors with **(A–C)** oligodendroglioma (WHO grade II; case 37, 52, 52) and **(D–F)** anaplastic oligodendroglioma (WHO grade III; case 44, 44, 57). GAL immunoreactivity is shown in **(A,D)**; GAL_1_-R immunoreactivity in **(B,E)**, and GAL_3_-R immunoreactivity in **(C,F)**. Arrow heads indicate positive-stained cells. [scale bar: 100 μm].

Overall, the cellular heterogeneity of glioma subtypes was reflected by a heterogeneous pattern of expression of GAL and GALRs (Figures 4–6, Table 1). The majority of gliomas showed focal GAL-immunoreactivity (71% of cases) and regions with diffuse GAL-staining (93% of cases). The proportion of GAL-positive stained cells ranged from <1 to 80%.

The most prominent receptor expressed in glioma was GAL_1_-R (29% of cases), followed by GAL_3_-R (24% of cases). GAL_2_-R was not detectable by IHC in glioma. The proportion of GAL_1_-R-stained cells as well as the proportion of GAL_3_-R-stained cells in the tissue sections was very low (mainly <1% of tumor cells).

Statistical testing revealed significant correlations between astrocytic, oligodendroglial and mixed neuronal-glial tumors in diffuse GAL staining (*p* = 0.009; Cramer-V). For focal GAL (*p* = 0.963; Cramer-V) as well as GAL_1_-R (*p* = 0.264; Cramer-V) and GAL_3_-R (*p* = 0.137; Cramer-V), no significant correlations between astrocytic, oligodendroglial, and mixed neuronal-glial tumors were observed.

Furthermore, correlations between GAL and GALR expression and different WHO grades I–IV were tested. The only significant correlation was found for GAL_3_-R and WHO grades (*p* = 0.015; Cramer-V): 13 of 55 samples were positive for GAL_3_-R, with 23% of GAL_3_-R positive samples being WHO grade I, 8% WHO grade II, 8% WHO grade III (39% WHO I–III) and 61% WHO grade IV, indicating that the significant correlation is between GAL_3_-R and WHO IV. Further testing of GAL_3_-R against WHO grade IV and non-WHO grade IV confirmed this presumption (*p* = 0.018; Fisher's exact test).

#### Expression of GAL and GALRs in Astrocytic Tumors

GAL-immunoreactivity showed substantial differences between and within different subtypes of 37 astrocytic tumors (WHO grade I–IV). In 70% of all subtypes, cases of focal GAL-immunoreactivity were observed, although the percentage of GAL-positive cells varied. Furthermore, in all astrocytic tumors, areas with diffuse GAL-staining were noticed (Figures 4, 5, Table 1, Supplementary Table 3). A small proportion of astrocytic tumors revealed expression of GAL_1_-R and GAL_3_-R, but only at low levels and in a small subset of tumor cells. Interestingly, GAL_2_-R-immunoreactivity was not detectable in astrocytic tumors.

Sixty percent of pilocytic astrocytomas (WHO grade I) displayed focal GAL-immunoreactivity (<1–18% GAL-positive tumor cells; Figure 4A, Table 1, Supplementary Table 3). In 57% of diffuse astrocytomas (WHO grade II), focal GAL-immunoreactivity (2–40% of tumor cells) was detected (Figure 4D). Also, 86% of anaplastic astrocytomas (WHO grade III) contained focal GAL-immunoreactivity (<1–65% of tumor cells; Figure 4G). In glioblastoma multiforme (WHO grade IV), GAL-positive cell staining was observed in 75% of cases (<1–30% of tumor cells, Figure 5A). In gliosarcoma (WHO grade IV), only half of the samples displayed focal GAL-immunoreactivity (15–70% of tumor cells, Figure 5D), whereas all giant cell glioblastomas (WHO grade IV) revealed focal GAL-immunoreactivity (35–80% of tumor cells; Figure 5G).

Only one out of 5 pilocytic astrocytoma (WHO grade I) showed GAL_1_-R-immunoreactivity in some tumor cells (<1% of tumor cells), with low staining intensity (Figure 4B). GAL_1_-R-immunoreactivity was not detectable in diffuse astrocytoma (WHO grade II; Figure 4E). About 43% of anaplastic astrocytomas (WHO grade III) revealed some weakly stained GAL_1_-R-positive cells (<1% of tumor cells; Figure 4H). In 38% of glioblastoma multiforme (WHO grade IV), GAL_1_-R-immunoreactivity was detected (<1–8% of tumor cells; Figure 5B). One third of gliosarcoma (WHO grade IV) and half of giant cell glioblastoma (WHO grade IV) samples were GAL_1_-R-positive (≤1% of tumor cells; Figures 5E,H).

A single pilocytic astrocytoma (WHO grade I) showed substantial GAL_3_-R expression in some tumor cells (<1% of tumor cells; Figure 4C). Tumor cell-associated GAL_3_-R-immunoreactivity was not detectable in diffuse astrocytoma (WHO grade II), anaplastic astrocytoma (WHO grade III) and giant cell glioblastoma (WHO grade IV; Figures 4F,I, 5I). In contrast, 63% of glioblastoma multiforme samples (WHO grade IV) and 50% of gliosarcomas (WHO grade IV) showed sparse GAL_3_-R-immunoreactivity (<1–7% of tumor cells; Figures 5C,F).

#### Expression of GAL and GALRs in Oligodendroglial Tumors

IHC analysis of 15 human oligodendroglial tumors (WHO grades II and III) revealed focal and diffuse GAL-immunoreactivity in the majority of cases. (Figure 6, Table 1, Supplementary Table 3). Receptor expression was generally low and found only in a subset of tumor cells. Tumor cell-associated GAL_2_-R-immunoreactivity was not detectable in oligodendroglial tumors.

In more detail, 78% of oligodendrogliomas (WHO grade II) showed focal GAL-immunoreactivity (<1–10% of tumor cells) and 89% contained areas of diffuse GAL staining (Figure 6A). Sixty percent of anaplastic oligodendrogliomas (WHO grade III) showed focal (<1–30% of tumor cells) and diffuse GAL-staining (Figure 6D).

GAL_1_-R-immunoreactivity was detectable in 22% of oligodendrogliomas (WHO grade II; <1% of tumor cells; Figure 6B). Anaplastic oligodendroglioma (WHO grade III) revealed sparse tumor cell-associated GAL_1_-R-immunoreactivity in 17% of samples (<1% of tumor cells; Figure 6E).

Tumor cell-associated GAL_3_-R-immunoreactivity was detectable in 11% of oligodendrogliomas (WHO grade II; <1% of tumor cells; Figure 6C). Sparse GAL_3_-R-immunoreactivity was detected in 17% of anaplastic oligodendrogliomas (WHO grade III; <1% of tumor cells; Figure 6F).

#### Expression of GAL and GALRs in Mixed Neuronal-Glial Tumors

**Figure 7:**
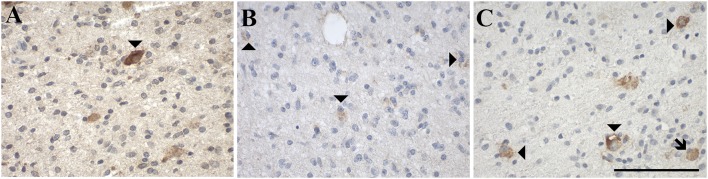
Representative images of immunohistochemical stainings of ganglioglioma (WHO grade I), a mixed neuronal-glial tumor, which show **(A)** GAL immunoreactivity (case 34), **(B)** GAL_1_-R immunoreactivity (case 36), and **(C)** GAL_3_-R immunoreactivity (case 34). Arrow heads indicate positive-stained cells whereas the arrow points out a GAL_3_-R positive GAM. [scale bar: 100 μm].

IHC analysis of three gangliogliomas (WHO grade I; Figure 7, Table 1, Supplementary Table 3) revealed focal (<1 and 6% of tumor cells) as well as diffuse GAL staining (Figure 7A) in two of the three cases. Interestingly one case was completely negative for focal and diffuse GAL staining.

GAL_1_-R-immunoreactivity was detectable in 67% of gangliogliomas (WHO grade I; ≤ 1% of tumor cells (Figure 7B).

Tumor cell-associated GAL_3_-R-immunoreactivity was detectable in 67% of gangliogliomas (WHO grade I; <1–3% of tumor cells; Figure 7C).

#### Expression of GAL and GALRs in Oligoastrocytic Tumors

IHC analysis of one oligoastrocytoma (OA, NOS; WHO grade II) and one anaplastic oligoastrocytoma (OAA, NOS; WHO grade III; Supplementary Table 3) revealed focal (20 and 90% of tumor cells) as well as diffuse GAL staining.

GAL_1_-R-immunoreactivity was detectable in both cases (WHO grade II and III; <1% of tumor cells).

Tumor cell-associated GAL_2_-R and GAL_3_-R-immunoreactivity was not detectable.

#### Expression of GAL and GALRs in Tumor-Infiltrating Immune Cells

**Figure 8:**
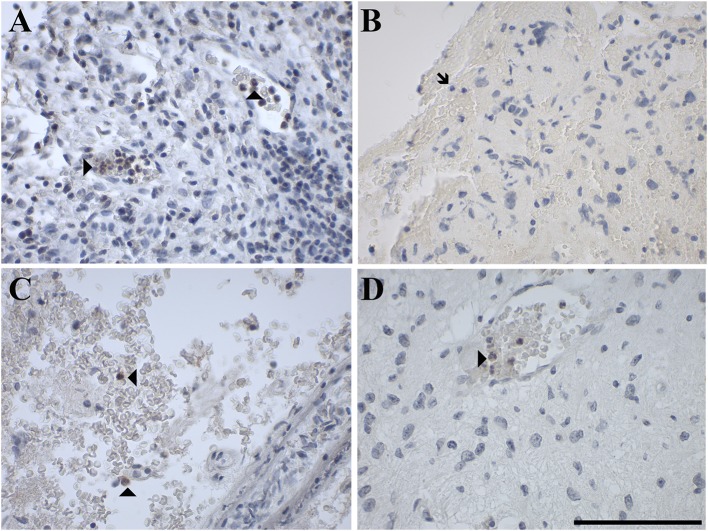
Representative images of immunohistochemical staining of neutrophil granulocytes stained positive for **(A)** GAL in a gliosarcoma (WHO grade IV; case 29), negative for **(B)** GAL_1_-R in a gliosarcoma (WHO grade IV; case 29) and positive for **(C)** GAL_2_-R in an anaplastic astrocytoma (WHO grade III; case 15) and **(D)** GAL_3_-R in a pilocytic astrocytoma (WHO grade I; case 5). Arrow heads indicate positive-stained neutrophil granulocytes, the arrow indicates a negative neutrophil granulocyte. [scale bar: 100 μm].

**Figure 9:**
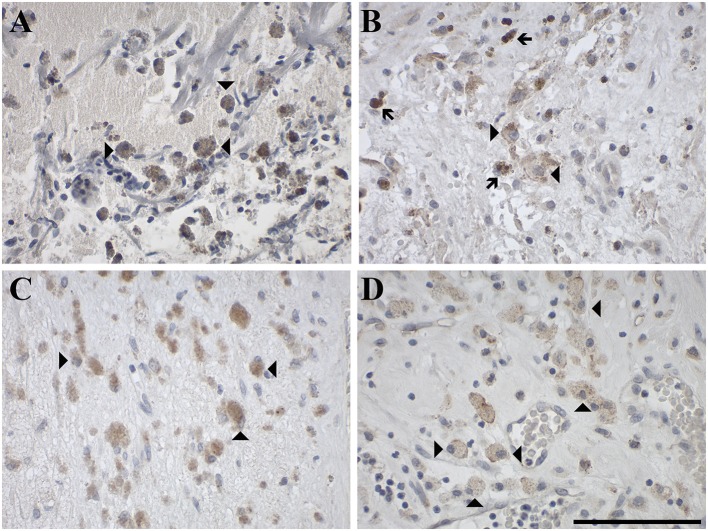
Representative images of immunohistochemical staining of GAMs stained positive for **(A)** GAL in an oligodendroglioma (WHO grade II; case 51), **(B)** GAL_1_-R in an anaplastic oligoastrocytoma, NOS III (case 54), **(C)** GAL_2_-R in an anaplastic oligoastrocytoma, NOS III (case 54) and **(D)** GAL_3_-R in a gliosarcoma (WHO grade IV; case 25). Arrow heads indicate positive-stained GAMs and arrows indicate hemosiderophages, which contain brown granules due to hemosiderin. [scale bar: 100 μm].

GAL and GALRs were also detected in infiltrating immune cells such a neutrophils and GAMs (Supplementary Tables 2, 3). GAL-immunoreactivity was sparse in neutrophilic granulocytes in glioma (5% of 57 cases) and was only observed in subpopulations (Figure 8). In 18% of glioma samples, GAMs were GAL positive (Figure 9). GAL-immunoreactivity was absent in tumor-associated immune cells in pituitary adenoma.

GAL_1_-R-immunoreactivity in GAMs was observed in 16% of glioma samples but was absent in neutrophils. Although GAL_2_-R-immunoreacitivity was undetectable in tumor cells of glioma and pituitary adenoma, 27% of glioma samples, particularly astrocytoma (WHO grade I-III), and 44% of pituitary adenomas revealed a small proportion of GAL_2_-R-positive neutrophilic granulocytes (Figure 8C). GAL_2_-R-positive GAMs were observed in one glioblastoma multiforme (WHO grade IV), one ganglioglioma (WHO grade I) and one anaplastic oligoastrocytoma (OAA, NOS; WHO grade III; Figure 9C).

**Figure 10:**
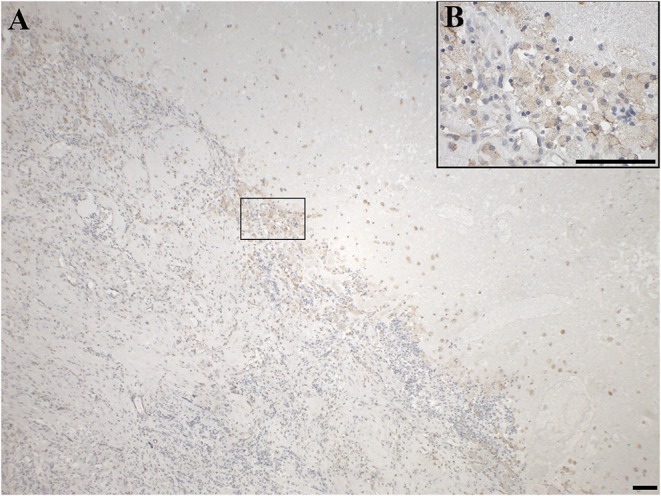
Immunohistochemical staining for GAL_3_-R of a gliosarcoma (WHO grade IV; case 25), showing positive GAMs at the junction of tumor tissue and necrotic tissue in **(A)** 4 × and **(B)** 40 × magnification. [scale bar: 100 μm].

GAL_3_-R was much more abundant in tumor-associated GAMs. In 35% of glioma samples, particularly high-grade glioma (WHO grade IV), GAL_3_-R-positive stained GAMs were identified (Figures 9D, 10). Forty nine percent of glioma samples and two pituitary adenomas revealed GAL_3_-R neutrophilic granulocytes (Figure 8D). GALR-positive GAMs were mainly localized near blood vessels or areas with necrotic tissue (Figure 10). GALR-positive neutrophilic granulocytes were mostly observed in hemorrhagic areas.

In addition to the presence of GAL_3_-R in immune cells, endothelial cells of blood vessels in glioma and pituitary adenoma showed frequent GAL_3_-R-immunoreactivity (Figure 5I, Supplementary Tables 2, 3). The other two receptors were not detected around blood vessels.

## Discussion

In this study, we show for the first time the cellular distribution of GALR proteins in human glioma, pituitary adenoma, and anterior pituitary gland.

Our findings correlate with our previous study, where GAL staining in glial cell bodies was found in 18 out of 20 (90%) human brain tumors, including glioblastoma multiforme (WHO grade IV), meningioma (WHO grade I-II), and gliosarcoma (WHO grade IV) (21). Presence of GALRs indicated by GAL binding occurred in 6 out of 15 gliomas (40%). In the present study, in 12 out of 18 (67%) gliomas (WHO grade IV), GALRs were detectable by IHC staining. The frequency of GALR-immunoreactivity in tumor cells was usually below 10% and the intensity of IHC staining was mainly low to medium, indicating low expression levels of the GALRs. Such low amounts of GALRs might be undetectable by GAL-binding studies. Due to the use of photoemulsions in binding studies using autoradiography, identification of the underlying cell type is not possible and therefore allocation of the binding to tumor cells, stroma or tumor-associated immune cells is not possible. Thus, we provide here the first evidence of GALR expression in tumor-infiltrating immune cells.

Previous RT-PCR analysis of glioblastoma multiforme (WHO grade IV) revealed that GAL_1_-R is the most prominent receptor, followed by GAL_3_-R and GAL_2_-R (21). This is in accordance with our study, which revealed GAL_1_-R and GAL_3_-R but not GAL_2_-R immunoreactivity. Low levels of the GALRs might be detectable by RT-PCR analysis but not IHC. This is also evident from our data in pituitary glands, where we detected all three GALRs by RT-PCR but only GAL_1_-R and GAL_3_-R by IHC staining.

Anti-proliferative effects of GAL and GAL_1_-R have been reported for HNSCC (35, 36, 40, 49). As we observed only minor amounts of GALR-positive tumor cell populations in glioma, downregulation of these receptors may be a survival mechanism of glioma cells to ensure proliferation. In accordance with this hypothesis, GAL suppressed proliferation of human U251 and T98G glioma cells via GAL_1_-R signaling (49).

Several studies reported GAL-like immunoreactivity in pituitary adenoma (11–16). The percentage of GAL-positive cases was dependent on the type of pituitary adenoma. Mainly ACTH-secreting pituitary adenomas were GAL-positive, whereas growth hormone- and prolactin-secreting as well as non-functioning pituitary adenomas showed lower frequencies of GAL-immunoreactivity. In ACTH-secreting adenoma, GAL seems to serve as a biomarker, with GAL levels being inversely correlated with tumor volume. Additionally, GAL-positive corticotroph adenomas were associated with a higher cure rate in patients (15), suggesting clinical relevance for GAL in this brain tumor type. In our study, 8 of 9 cases of pituitary adenoma had at least some areas with diffuse GAL-immunoreactivity. Our data on the expression of GAL in pituitary adenoma (Supplementary Table 2) correlate with previous published data, where 77% of patients with Cushing's disease (ACTH-secreting), 25% of patients with acromegaly (growth hormone-secreting), 13% of prolactinomas and 34% of non-functioning tumors expressed GAL (Supplementary Table 5).

Corresponding to observations in glioma samples, receptor downregulation might also be a possible mechanism in pituitary adenoma to escape anti-proliferative effects of GAL. While all pituitary gland samples expressed GAL_1_-R and GAL_3_-R, GALR expression was absent in pituitary adenoma. Furthermore, it also seemed that the tumor itself reduced GAL expression, as only 11% of pituitary adenoma showed focal GAL staining, in contrast to 100% of healthy pituitary glands. However, it cannot be ruled out that the observations in pituitary glands and pituitary adenoma are also age-related effects, as the median age of the groups was different.

In general, we observed focal as well as diffuse GAL-like immunoreactivity in human brain tumors. Diffuse GAL staining is representative of secreted GAL peptide being present extracellularly. However, it cannot be determined whether the secreted GAL originates either from the cells in the near vicinity showing focal GAL staining or from other brain regions. Additionally, it is unclear whether the secreted GAL originates from the tumor itself or from adjacent healthy tissue, for example, the pituitary which was shown to exhibit high GAL mRNA levels as well as medium to strong diffuse and focal GAL staining.

The expression of the galanin system analyzed by RT-PCR revealed case-dependent expression patterns of GAL and GALR mRNA (34). As already discussed above, RT-PCR analysis is more sensitive than IHC and this could partially explain why GALRs were not detectable in tumor cells of our pituitary adenomas by IHC analysis. As RNA is isolated from whole tumor extracts, expression of the receptors in non-tumor cells will also be detected. The level of GALRs expressing immune-infiltrating cells might at least partially account for the case differences in GALR mRNA expression levels in pituitary adenoma (34). Furthermore, GAL-immunoreactivity was much lower in pituitary adenoma as in the anterior pituitary gland. This is in accordance with GAL mRNA expression data, which showed lower GAL expression levels in 10 out of 13 pituitary adenomas (34).

The expression of GAL_3_-R in small blood vessels is in agreement with the expression of GAL_3_-R in the skin vasculature (48).

GAMs are key drivers of the local immunosuppressive microenvironment that promotes tumor progression and tumor resistance to immunomodulating therapeutics. Together with other myeloid cells, such as dendritic cells and neutrophils, GAMs actively shape glioma development and the glioma microenvironment, modulate the anti-tumoral immune response, and support angiogenesis, tumor cell invasion and proliferation (50). Therefore, it could also be possible that GAL is secreted by the tumor cells showing focal GAL staining to boost tumor-supporting properties of the GAMs.

GAL and GALR mRNA expression has already been reported for immune cells isolated from peripheral blood. Macrophages express and secrete substantial amounts of galanin (7). However, GAMs showed no detectable GAL-immunoreactivity. In contrast, the majority of GAMs were GAL_3_-R positive. This high proportion of GAL_3_-R-immunoreactivity in GAMs is in contrast to the expression of GALRs in peripheral macrophages. A xanthelasma, also referred to as xanthoma, is a cluster of foam cells in the connective tissue of the skin. The foam cells are formed by macrophages accumulating lipids by phagocytosis (51). We reported membrane-associated GAL_1_-R as well as GAL_2_-R staining on some macrophages in the xanthelasma deposits (7). To our knowledge, there are no other studies available on the expression of GALRs in tumor-associated macrophages and therefore it is not known if the expression of GALRs is restricted to GAMs or if this is also the case in tumor-associated macrophages of other tumor entities. In addition, we are not aware of any study reporting the expression of the GAL system, especially GAL_3_-R, in tumor-associated neutrophilic granulocytes.

Interestingly, in our previous studies we observed that GAL can have pro- and anti-inflammatory properties on macrophage function depending on their differentiation and polarization status (7). Therefore, it could be possible that GAL also induces tumor-suppressing functions in GAL_3_-R positive GAMs. Regarding immunity and inflammation, we reported recently that GAL_3_-R signaling has both pro- and anti-inflammatory properties (48, 52).

Currently, possible treatment strategies targeting GALR subtypes are still hampered by the lack of single-subtype specific agonists or antagonists [for review see (6)]. Most available selective ligands are peptidergic compounds, which makes their clinical application problematic due to peptide degradation. Furthermore, a GAL_3_-R-specific non-peptidergic antagonist is available, but we showed non-GALR-mediated toxicity of this compound (53). Therefore, there is a need to develop novel selective, stable and non-peptidergic GALR ligands.

In conclusion, our data indicate that GALR signaling could influence the behavior of tumor cell-associated immune cells. Future studies should focus on the characterization of the immune cell subtypes expressing GALRs in glioma, for example by using markers to differentiate GAMs into resident microglia and bone marrow-derived macrophages. Based on the size and shape of the cells and their nuclei, GALR-positive GAMs resemble the bone marrow-derived macrophage type. Recently it has been shown that GAL is able to influence immune cell behavior by modulating cytokine expression and release, as demonstrated in human neutrophils, natural killer cells, monocytes and macrophages (7, 48, 54, 55). Furthermore, galanin down-regulates microglial tumor necrosis factor-alpha production and induces microglial migration (56, 57). Thus, strategies targeting tumor-supportive myeloid cells represent an encouraging novel therapeutic approach and could also be considered for the GAL system.

## Data Availability Statement

The raw data supporting the conclusions of this article will be made available by the authors, without undue reservation, to any qualified researcher.

## Ethics Statement

Ethical review and approval was not required for the study on human participants in accordance with the local legislation and institutional requirements. Written informed consent to participate in this study was provided by the participants' legal guardian/next of kin.

## Author Contributions

SF and JL designed and performed experiments. SF, JL, SB, and TR analyzed data. SF, SB, and BK contributed to drafting the manuscript. SW collected and/or provided human patient samples, rendered the neuropathology diagnoses, co-edited the manuscript, and critically discussed the data. BK obtained resources for the study, designed experiments, critically discussed the data, and co-edited the manuscript. All authors approved the final version of the manuscript.

### Conflict of Interest

The authors declare that the research was conducted in the absence of any commercial or financial relationships that could be construed as a potential conflict of interest.

## References

[1] Ostrom QT Gittleman H de Blank PM Finlay JL Gurney JG McKean-Cowdin R. American brain tumor association adolescent and young adult primary brain and central nervous system tumors diagnosed in the United States in 2008-2012. Neuro Oncol. (2016) 18:i1–50. 10.1093/neuonc/nov297 26705298 PMC4690545

[2] Louis DN Perry A Reifenberger G von Deimling A Figarella-Branger D Cavenee WK. The 2016 world health organization classification of tumors of the central nervous system: a summary. Acta Neuropathol. (2016) 131:803–20. 10.1007/s00401-016-1545-1 27157931

[3] Sethi T Langdon S Smyth J Rozengurt E. Growth of small cell lung cancer cells: stimulation by multiple neuropeptides and inhibition by broad spectrum antagonists *in vitro* and *in vivo*. Cancer Res. (1992) 52(Suppl. 9):2737s–42. 1314136

[4] Cochaud S Chevrier L Meunier AC Brillet T Chadeneau C Muller JM. The vasoactive intestinal peptide-receptor system is involved in human glioblastoma cell migration. Neuropeptides. (2010) 44:373–83. 10.1016/j.npep.2010.06.003 20638719

[5] Cochaud S Meunier AC Monvoisin A Bensalma S Muller JM Chadeneau C. Neuropeptides of the VIP family inhibit glioblastoma cell invasion. J Neurooncol. (2015) 122:63–73. 10.1007/s11060-014-1697-6 25563813

[6] Lang R Gundlach AL Holmes FE Hobson SA Wynick D Hokfelt T. Physiology, signaling, and pharmacology of galanin peptides and receptors: three decades of emerging diversity. Pharmacol Rev. (2015) 67:118–75. 10.1124/pr.112.006536 25428932

[7] Koller A Brunner SM Bianchini R Ramspacher A Emberger M Locker F. Galanin is a potent modulator of cytokine and chemokine expression in human macrophages. Sci Rep. (2019) 9:7237. 10.1038/s41598-019-43704-7 31076613 PMC6510899

[8] Bauer FE Hacker GW Terenghi G Adrian TE Polak JM Bloom SR. Localization and molecular forms of galanin in human adrenals: elevated levels in pheochromocytomas. J Clin Endocrinol Metab. (1986) 63:1372–8. 10.1210/jcem-63-6-1372 2430990

[9] Hacker GW Bishop AE Terenghi G Varndell IM Aghahowa J Pollard K. Multiple peptide production and presence of general neuroendocrine markers detected in 12 cases of human phaeochromocytoma and in mammalian adrenal glands. Virchows Arch A Pathol Anat Histopathol. (1988) 412:399–411. 10.1007/BF00750574 3128912

[10] Hulting AL Meister B Grimelius L Wersall J Anggard A Hökfelt T. Production of a galanin-like peptide by a human pituitary adenoma: immunohistochemical evidence. Acta Physiol Scand. (1989) 137:561–2. 10.1111/j.1748-1716.1989.tb08801.x 2481382

[11] Vrontakis ME Sano T Kovacs K Friesen HG. Presence of galanin-like immunoreactivity in nontumorous corticotrophs and corticotroph adenomas of the human pituitary. J Clin Endocrinol Metab. (1990) 70:747–51. 10.1210/jcem-70-3-747 1689739

[12] Bennet WM Hill SF Ghatei MA Bloom SR. Galanin in the normal human pituitary and brain and in pituitary adenomas. J Endocrinol. (1991) 130:463–7. 10.1677/joe.0.1300463 1719117

[13] Hsu DW Hooi SC Hedley-Whyte ET Strauss RM Kaplan LM. Coexpression of galanin and adrenocorticotropic hormone in human pituitary and pituitary adenomas. Am J Pathol. (1991) 138:897–909. 1707237 PMC1886121

[14] Sano T Vrontakis ME Kovacs K Asa SL Friesen HG. Galanin immunoreactivity in neuroendocrine tumors. Arch Pathol Lab Med. (1991) 115:926–9. 1718239

[15] Leung B Iisma TP Leung KC Hort YJ Turner J Sheehy JP. Galanin in human pituitary adenomas: frequency and clinical significance. Clin Endocrinol. (2002) 56:397–403. 10.1046/j.1365-2265.2002.01486.x 11940053

[16] Grenback E Bjellerup P Wallerman E Lundblad L Anggard A Ericson K. Galanin in pituitary adenomas. Regul Pept. (2004) 117:127–39. 10.1016/j.regpep.2003.10.022 14700749

[17] Felix I Bilbao JM Asa SL Tyndel F Kovacs K Becker LE. Cerebral and cerebellar gangliocytomas: a morphological study of nine cases. Acta Neuropathol. (1994) 88:246–51. 10.1007/BF00293400 7810295

[18] Fried G Wikstrom LM Hoog A Arver S Cedermark B Hamberger B. Multiple neuropeptide immunoreactivities in a renin-producing human paraganglioma. Cancer. (1994) 74:142–51. 10.1002/1097-0142(19940701)74:1<142::aid-cncr2820740123>3.0.co;2-o 7911735

[19] Tadros TS Strauss RM Cohen C Gal AA. Galanin immunoreactivity in paragangliomas but not in carcinoid tumors. Appl Immunohistochem Mol Morphol. (2003) 11:250–2. 10.1097/00129039-200309000-00008 12966352

[20] Tuechler C Hametner R Jones N Jones R Iismaa TP Sperl W. Galanin and galanin receptor expression in neuroblastoma. Ann N Y Acad Sci. (1998) 863:438–41. 10.1111/j.1749-6632.1998.tb10718.x 9928194

[21] Berger A Santic R Almer D Hauser-Kronberger C Huemer M Humpel C. Galanin and galanin receptors in human gliomas. Acta Neuropathol. (2003) 105:555–60. 10.1007/s00401-003-0680-7 12734662

[22] Gilaberte Y Vera J Coscojuela C Roca MJ Parrado C Gonzalez S. Expression of galanin in melanocytic tumors. Actas Dermosifiliogr. (2007) 98:24–34. 10.1016/S1578-2190(07)70386-4 17374330

[23] Sugimoto T Seki N Shimizu S Kikkawa N Tsukada J Shimada H. The galanin signaling cascade is a candidate pathway regulating oncogenesis in human squamous cell carcinoma. Genes Chromoso Cancer. (2009) 48:132–42. 10.1002/gcc.20626 18973137

[24] Kepron C Reis P Bharadwaj R Shaw J Kamel-Reid S Ghazarian D. Identification of genomic predictors of non-melanoma skin cancer in solid organ transplant recipients. Eur J Dermatol. (2009) 19:278–80. 10.1684/ejd.2009.0649 19307168

[25] Kim KY Kee MK Chong SA Nam MJ. Galanin is up-regulated in colon adenocarcinoma. Cancer Epidemiol Biomark Prev. (2007) 16:2373–8. 10.1158/1055-9965.EPI-06-0740 18006926

[26] Godlewski J Pidsudko Z. Characteristic of galaninergic components of the enteric nervous system in the cancer invasion of human large intestine. Ann Anat. (2012) 194:368–72. 10.1016/j.aanat.2011.11.009 22226150

[27] Stevenson L Allen WL Turkington R Jithesh PV Proutski I Stewart G. Identification of galanin and its receptor GalR1 as novel determinants of resistance to chemotherapy and potential biomarkers in colorectal cancer. Clin Cancer Res. (2012) 18:5412–26. 10.1158/1078-0432.CCR-12-1780 22859720 PMC3463501

[28] Skotheim RI Lind GE Monni O Nesland JM Abeler VM Fossa SD. Differentiation of human embryonal carcinomas *in vitro* and *in vivo* reveals expression profiles relevant to normal development. Cancer Res. (2005) 65:5588–98. 10.1158/0008-5472.CAN-05-0153 15994931

[29] Hulting AL Land T Berthold M Langel U Hökfelt T Bartfai T. Galanin receptors from human pituitary tumors assayed with human galanin as ligand. Brain Res. (1993) 625:173–6. 10.1016/0006-8993(93)90152-D 7694774

[30] Berger A Santic R Hauser-Kronberger C Schilling FH Kogner P Ratschek M. Galanin and galanin receptors in human cancers. Neuropeptides. (2005) 39:353–9. 10.1016/j.npep.2004.12.016 15944034

[31] Misawa K Ueda Y Kanazawa T Misawa Y Jang I Brenner JC. Epigenetic inactivation of galanin receptor 1 in head and neck cancer. Clin Cancer Res. (2008) 14:7604–13. 10.1158/1078-0432.CCR-07-4673 19047085 PMC3189853

[32] Berger A Tuechler C Almer D Kogner P Ratschek M Kerbl R. Elevated expression of galanin receptors in childhood neuroblastic tumors. Neuroendocrinology. (2002) 75:130–8. 10.1159/000048229 11867941

[33] Perel Y Amrein L Dobremez E Rivel J Daniel JY Landry M. Galanin and galanin receptor expression in neuroblastic tumours: correlation with their differentiation status. Br J Cancer. (2002) 86:117–22. 10.1038/sj.bjc.6600019 11857022 PMC2746536

[34] Tofighi R Barde S Palkovits M Hoog A Hokfelt T Ceccatelli S. Galanin and its three receptors in human pituitary adenoma. Neuropeptides. (2012) 46:195–201. 10.1016/j.npep.2012.07.003 22889491

[35] Henson BS Neubig RR Jang I Ogawa T Zhang Z Carey TE. Galanin receptor 1 has anti-proliferative effects in oral squamous cell carcinoma. J Biol Chem. (2005) 280:22564–71. 10.1074/jbc.M414589200 15767248

[36] Kanazawa T Iwashita T Kommareddi P Nair T Misawa K Misawa Y. Galanin and galanin receptor type 1 suppress proliferation in squamous carcinoma cells: activation of the extracellular signal regulated kinase pathway and induction of cyclin-dependent kinase inhibitors. Oncogene. (2007) 26:5762–71. 10.1038/sj.onc.1210384 17384686

[37] Berger A Lang R Moritz K Santic R Hermann A Sperl W. Galanin receptor subtype GalR2 mediates apoptosis in SH-SY5Y neuroblastoma cells. Endocrinology. (2004) 145:500–7. 10.1210/en.2003-0649 14592962

[38] Tofighi R Joseph B Xia S Xu ZQ Hamberger B Hökfelt T. Galanin decreases proliferation of PC12 cells and induces apoptosis via its subtype 2 receptor (GalR2). Proc Natl Acad Sci USA. (2008) 105:2717–22. 10.1073/pnas.0712300105 18272487 PMC2268202

[39] Kanazawa T Kommareddi PK Iwashita T Kumar B Misawa K Misawa Y. Galanin receptor subtype 2 suppresses cell proliferation and induces apoptosis in p53 mutant head and neck cancer cells. Clin Cancer Res. (2009) 15:2222–30. 10.1158/1078-0432.CCR-08-2443 19276245 PMC3315370

[40] Kanazawa T Misawa K Misawa Y Maruta M Uehara T Kawada K. Galanin receptor 2 utilizes distinct signaling pathways to suppress cell proliferation and induce apoptosis in HNSCC. Mol Med Rep. (2014) 10:1289–94. 10.3892/mmr.2014.2362 25017118

[41] Sethi T Rozengurt E. Galanin stimulates Ca2+ mobilization, inositol phosphate accumulation, and clonal growth in small cell lung cancer cells. Cancer Res. (1991) 51:1674–9. 1705478

[42] Roelle S Grosse R Buech T Chubanov V Gudermann T. Essential role of Pyk2 and Src kinase activation in neuropeptide-induced proliferation of small cell lung cancer cells. Oncogene. (2008) 27:1737–48. 10.1038/sj.onc.1210819 17906699

[43] Schrodl F Kaser-Eichberger A Trost A Strohmaier C Bogner B Runge C. Distribution of galanin receptors in the human eye. Exp Eye Res. (2015) 138:42–51. 10.1016/j.exer.2015.06.024 26122049

[44] Louis DN Ohgaki H Wiestler OD Cavenee WK Ellison DW Figarella-Branger D. WHO Classification of Tumours of the Central Nervous System. Lyon: IARC (2016). 10.1007/s00401-016-1545-1 27157931

[45] Brunner SM Koller A Stockinger J Locker F Leis S Ernst F. Validation of antibody-based tools for galanin research. Peptides. (2019) 120:170009. 10.1016/j.peptides.2018.08.010 30196126

[46] Kofler B Berger A Santic R Moritz K Almer D Tuechler C. Expression of neuropeptide galanin and galanin receptors in human skin. J Invest Dermatol. (2004) 122:1050–3. 10.1111/j.0022-202X.2004.22418.x 15102097

[47] Bovell DL Holub BS Odusanwo O Brodowicz B Rauch I Kofler B. Galanin is a modulator of eccrine sweat gland secretion. Exp Dermatol. (2013) 22:141–3. 10.1111/exd.12067 23278944

[48] Locker F Vidali S Holub BS Stockinger J Brunner SM Ebner S. Lack of galanin receptor 3 alleviates psoriasis by altering vascularization, immune cell infiltration, and cytokine expression. J Invest Dermatol. (2018) 138:199–207. 10.1016/j.jid.2017.08.015 28844939

[49] Mei Z Yang Y Li Y Yang F Li J Xing N. Galanin suppresses proliferation of human U251 and T98G glioma cells via its subtype 1 receptor. Biol Chem. (2017) 398:1127–39. 10.1515/hsz-2016-0320 28525358

[50] Locarno CV Simonelli M Carenza C Capucetti A Stanzani E Lorenzi E. Role of myeloid cells in the immunosuppressive microenvironment in gliomas. Immunobiology. (2019) 225:151853. 10.1016/j.imbio.2019.10.002 31703822

[51] Zak A Zeman M Slaby A Vecka M. Xanthomas: clinical and pathophysiological relations. Biomed Pap Med Fac Univ Palacky Olomouc Czech Repub. (2014) 158:181–8. 10.5507/bp.2014.016 24781043

[52] Botz B Kemeny A Brunner SM Locker F Csepregi J Mocsai A. Lack of galanin 3 receptor aggravates murine autoimmune arthritis. J Mol Neurosci. (2016) 59:260–9. 10.1007/s12031-016-0732-9 26941032 PMC4884566

[53] Koller A Rid R Beyreis M Bianchini R Holub BS Lang A. In vitro toxicity of the galanin receptor 3 antagonist SNAP 37889. Neuropeptides. (2016) 56:83–8. 10.1016/j.npep.2015.12.003 26725588

[54] Koller A Bianchini R Schlager S Munz C Kofler B Wiesmayr S. The neuropeptide galanin modulates natural killer cell function. Neuropeptides. (2017) 64:109–15. 10.1016/j.npep.2016.11.002 27837916

[55] Ramspacher A Neudert M Koller A Schlager S Kofler B Brunner SM. Influence of the regulatory peptide galanin on cytokine expression in human monocytes. Ann N Y Acad Sci. (2019) 1455:185–95. 10.1111/nyas.14111 31074091 PMC6899851

[56] Su Y Ganea D Peng X Jonakait GM. Galanin down-regulates microglial tumor necrosis factor-alpha production by a post-transcriptional mechanism. J Neuroimmunol. (2003) 134:52–60. 10.1016/S0165-5728(02)00397-1 12507772

[57] Ifuku M Okuno Y Yamakawa Y Izumi K Seifert S Kettenmann H. Functional importance of inositol-1,4,5-triphosphate-induced intracellular Ca2+ mobilization in galanin-induced microglial migration. J Neurochem. (2011) 117:61–70. 10.1111/j.1471-4159.2011.07176.x 21226711

