# Supplementary material for: Galanin System in Human Glioma and Pituitary Adenoma
Source: Front Endocrinol (Lausanne). 2020 Mar 24;11:155. doi: 10.3389/fendo.2020.00155 (PMC7105811; doi:10.3389/fendo.2020.00155)
Supplement: Supplementary file 1 [file Data_Sheet_1.docx]

Supplementary Material

# Supplementary Figures


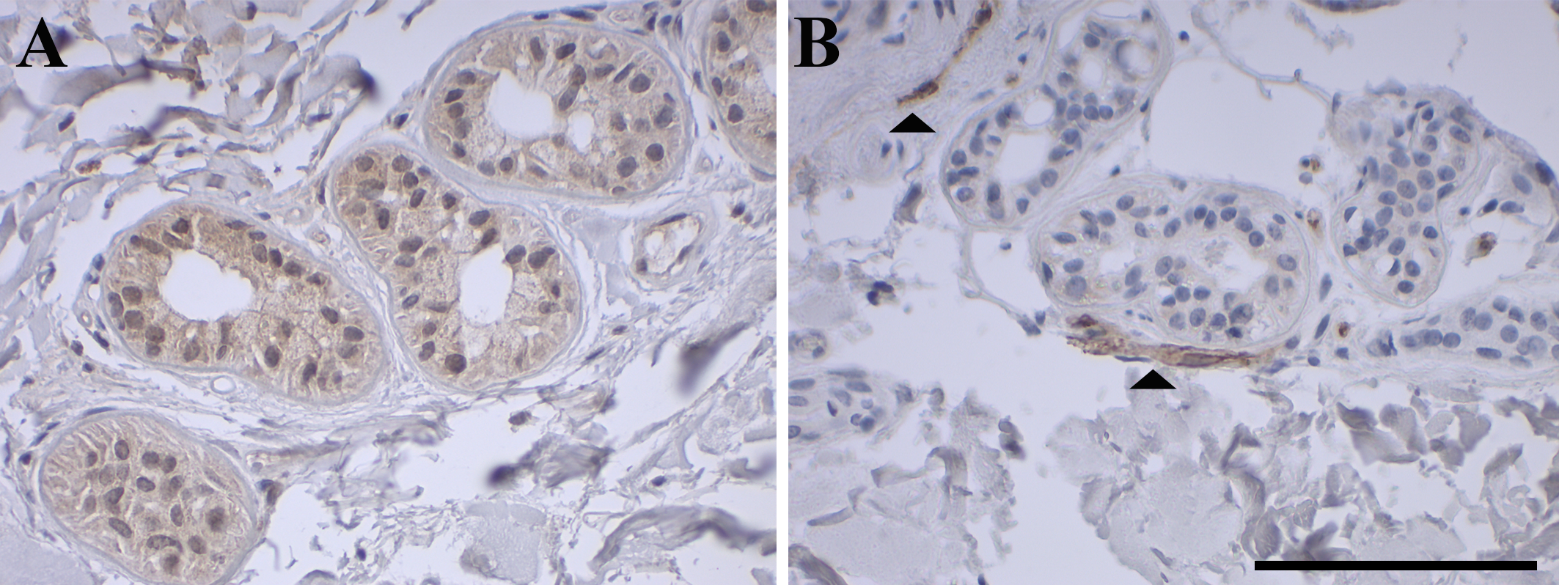


**SUPPLEMENTARY FIGURE 1|** Representative images of immunohistochemical staining of human skin as positive controls for (**A**) GAL-immunoreactivity showing positive sweat glands and (**B**) GAL_3_-R-immunoreactivity showing positive blood vessels. [scale bar: 100 μm]


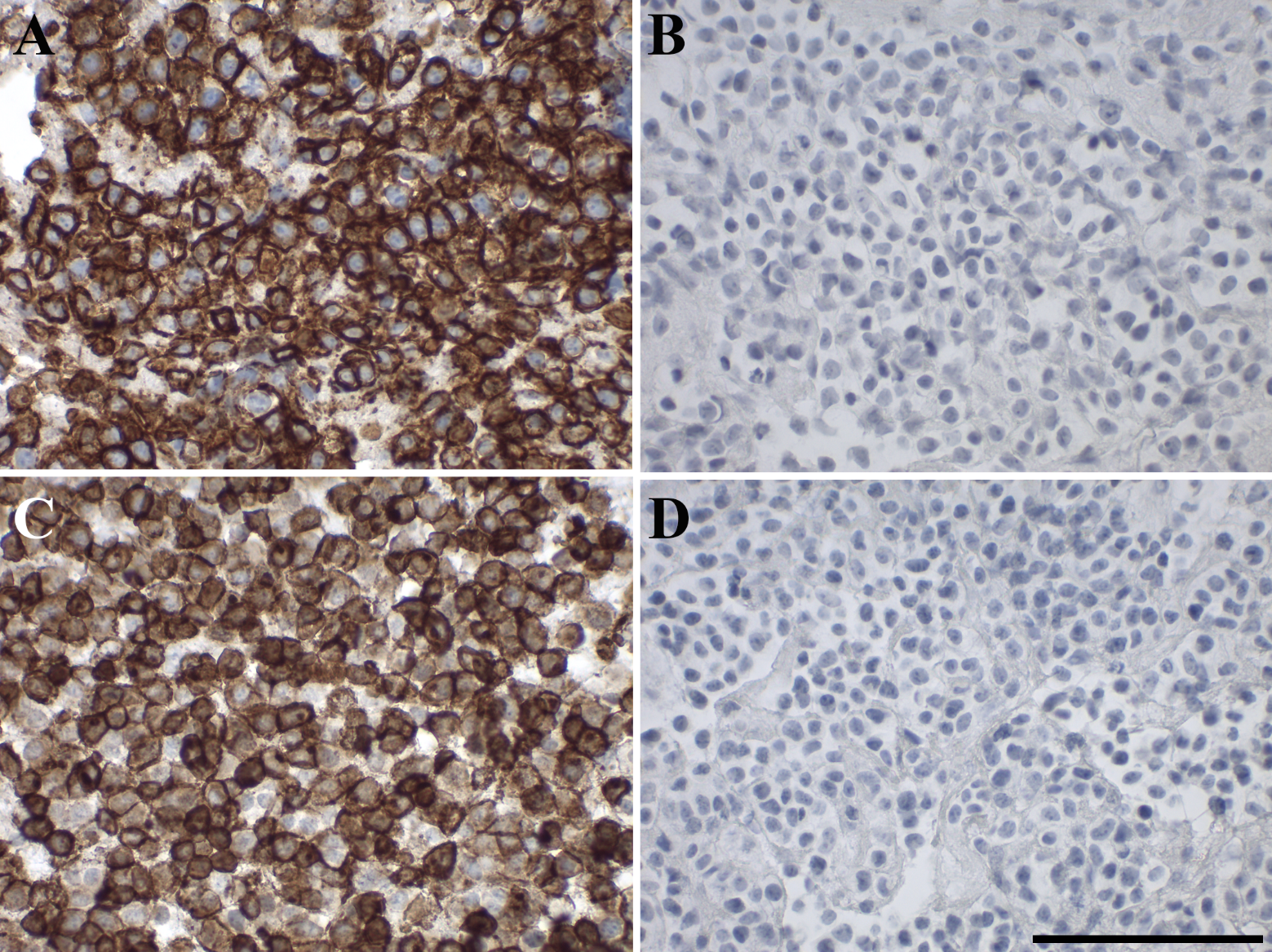


**SUPPLEMENTARY FIGURE 2|** Representative images of immunohistochemical staining of SH-SY5Y cells transfected with **(A**, **B)** GAL_1_-R or **(C**, **D)** GAL_2_-R as positive controls for (**A**) GAL_1_-R-immunoreactivity and (**C**) GAL_2_-R-immunoreactivity. (**B**, **D**) Secondary antibody only control staining. [scale bar: 100 μm]

# Supplementary Tables

**SUPPLEMENTARY TABLE 1|** Detailed information on anterior pituitary glands. Besides general information like disease and age [years] of the patients, the density [%] and intensity [0-3] of GAL and GALR immunoreactivity are given. Additionally, positive blood vessels are indicated. For GAL_2_-R no density [%] and intensity [0-3] values are given, since all samples were negative for GAL_2_-R immunoreactivity.

|  |  |  | **GAL** | | |  | **GAL_1_-R** | |  | **GAL_3_-R** | | |
| --- | --- | --- | --- | --- | --- | --- | --- | --- | --- | --- | --- | --- |
| **Case** | **Age**  **[years]** |  | **Dens [%]** | **Int** | **DS** |  | **Dens [%]** | **Int** |  | **Dens [%]** | **Int** | **BV** |
| **Anterior Pituitary Glands** | | | | | | | | | | | | |
| 1 | 66 |  | 20-25 | 3 | 1-2 |  | 15 | 3 |  | 3 | 2-3 | + |
| 2 | 61 |  | 2 | 3 | 1-2 |  | 10 | 3 |  | <1 | 2-3 | + |
| 3 | 92 |  | 40 | 3 | 1-2 |  | 15 | 3 |  | 2 | 2-3 | + |
| 4 | 81 |  | 2 | 3 | 1-2 |  | 7 | 3 |  | 5 | 2-3 | + |
| 5 | 78 |  | 2 | 3 | 1-2 |  | 12 | 3 |  | 1 | 2-3 | + |
| 6 | 58 |  | 5-10 | 3 | 1-2 |  | 15 | 3 |  | <1 | 2-3 | + |
| 7 | 92 |  | 15 | 3 | 1-2 |  | 15 | 3 |  | 4 | 2-3 | + |

Dens, Density; Int, Intensity; DS, diffuse staining; BV, blood vessels.

**SUPPLEMENTARY TABLE 2|** Detailed information on each case of pituitary adenoma. Besides general information like the disease and age [years] of the patients, the density [%] and intensity [0-3] of GAL-immunoreactivity are given. Additionally, positive neutrophil granulocytes, GAMs and blood vessels are indicated. For GAL_1_-R, GAL_2_-R and GAL_3_-R, no density [%] and intensity [0-3] values are given, since all samples were negative for the respective immunoreactivity in tumor cells.

|  |  |  |  | **GAL** | | | | | **GAL_2_-R** | | | | **GAL_3_-R** | | | |
| --- | --- | --- | --- | --- | --- | --- | --- | --- | --- | --- | --- | --- | --- | --- | --- | --- |
| **Case** | **Hormone status** | **Age [years]** |  | **Dens [%]** | **Int** | **DS** | **N** | **M** |  | **N** | **M** |  | **N** | **M** | **BV** | |
| **Pituitary Adenoma** | | | | | | | | | | | | | | | |  |
| 1 | prolactin | 62 |  | 0 | 0 | 0 |  |  |  | + |  |  | + |  | + | |
| 2 | null cell | 74 |  | 0 | 0 | 0-1 |  |  |  |  |  |  |  |  | + | |
| 3 | FSH | 73 |  | 0 | 0 | 0-2 |  |  |  | + |  |  | + |  | + | |
| 4 | FSH | 70 |  | 0 | 0 | 0-1 |  |  |  |  |  |  |  |  |  | |
| 5 | null cell | 57 |  | 0 | 0 | 0-1 |  |  |  |  |  |  |  |  |  | |
| 6 | STH, prolactin | 38 |  | 0 | 0 | 0-2 |  |  |  |  |  |  |  |  |  | |
| 7 | null cell | 51 |  | 2 | 2-3 | 0-2 |  |  |  | + |  |  |  |  | + | |
| 8 | TSH, STH, prolactin | 27 |  | 0 | 0 | 0-2 |  |  |  |  |  |  |  |  | + | |
| 9 | prolactin | 54 |  | 0 | 0 | 0-2 |  |  |  | + |  |  |  |  |  | |

Dens, Density; Int, Intensity; DS, diffuse staining; N, neutrophil granulocytes; M, glioma-associated macrophages and microglia ; BV, blood vessels; FSH, follicle-stimulating hormone; STH, somatotropin hormone; TSH, thyroid-stimulating hormone.

**SUPPLEMENTARY TABLE 3|** Detailed information on each case of glioma. Besides general information like the disease and age [years] of all cases, the density [%] and intensity [0-3] of GAL and GALR immunoreactivity are given. Additionally, positive neutrophil granulocytes, GAMs and blood vessels are indicated. For GAL_2_-R, no density [%] and intensity [0-3] values are given, since all samples were negative for GAL_2_-R immunoreactivity.

|  |  |  | **GAL** | | | | |  | **GAL_1_-R** | | | | **GAL_2_-R** | | | | **GAL_3_-R** | | | | |
| --- | --- | --- | --- | --- | --- | --- | --- | --- | --- | --- | --- | --- | --- | --- | --- | --- | --- | --- | --- | --- | --- |
| **Case** | **Age**  **[years]** |  | **Dens [%]** | **Int** | **DS** | **N** | **M** |  | **Dens [%]** | **Int** | **N** | **M** |  | **N** | **M** |  | **Dens [%]** | **Int** | **N** | **M** | **BV** |
| **Pilocytic astrocytoma I** | | | | | | | | | | | | | | | | | | | | | |
| 1 | 3 |  | 18 | 2-3 | 2 |  |  |  | <1 | 1 |  |  |  | + |  |  | 0 | 0 | + |  | + |
| 2 | 12 |  | 1 | 2 | 1-2 | + | + |  | 0 | 0 |  |  |  |  |  |  | 0 | 0 | + |  | + |
| 3 | 21 |  | 10 | 1-2 | 1 |  |  |  | 0 | 0 |  |  |  | + |  |  | 0 | 1-2 | + |  | + |
| 4 | 16 |  | 0 | 0 | 0-1 |  |  |  | 0 | 0 |  |  |  | + |  |  | <1 | 2-3 | + | + | + |
| 5 | 17 |  | 0 | 0 | 0-1 | + |  |  | 0 | 0 |  |  |  |  |  |  | 0 | 0 | + |  |  |
| **Diffuse astrocytoma II** | | | | | | | | | | | | | | | | | | | | | |
| 6 | 4 |  | 8 | 2-3 | 1-2 |  |  |  | 0 | 0 |  |  |  |  |  |  | 0 | 1 | + |  |  |
| 7 | 31 |  | 0 | 0 | 1-2 |  |  |  | 0 | 0 |  |  |  |  |  |  | 0 | 0 |  |  |  |
| 8 | 76 |  | 40 | 2 | 0-2 |  |  |  | 0 | 0 |  |  |  |  |  |  | 0 | 0 |  |  |  |
| 9 | 24 |  | 7 | 2 | 0-2 |  | + |  | 0 | 0 |  |  |  | + |  |  | 0 | 0 | + |  |  |
| 10 | 46 |  | 2 | 1-2 | 0-1 |  |  |  | 0 | 0 |  |  |  | + |  |  | 0 | 0 | + |  | + |
| 49 | 31 |  | 0 | 0 | 0-2 |  |  |  | 0 | 0 |  |  |  |  |  |  | 0 | 0 |  |  |  |
| 53 | 44 |  | 0 | 1-2 | 0-1 |  | + |  | 0 | 0 |  |  |  | + |  |  | 0 | 0 | + |  |  |
| **Anaplastic astrocytoma III** | | | | | | | | | | | | | | | | | | | | | |
| 11 | 4 |  | 65 | 1-2 | 1 |  |  |  | 0 | 0 |  |  |  |  |  |  | 0 | 0 |  |  |  |
| 12 | 33 |  | 27 | 1-2 | 1 |  |  |  | <1 | 1 |  |  |  |  |  |  | 0 | 0 |  | + |  |
| 13 | 62 |  | 15 | 1-2 | 1 |  |  |  | 0 | 1 |  | + |  |  |  |  | 0 | 0 | + |  | + |
| 14 | 24 |  | 2 | 1-2 | 0-2 |  |  |  | 0 | 0 |  |  |  | + |  |  | 0 | 0 | + |  | + |
| 15 | 33 |  | 0 | 0 | 0-2 |  |  |  | 0 | 0 |  |  |  | + |  |  | 0 | 0 | + |  |  |
| 55 (rec) | 71 |  | 50 | 1-2 | 1-2 |  |  |  | <1 | 1 |  | + |  | + |  |  | 0 | 0 |  | + | + |
| 56 | 60 |  | <1 | 1-2 | 0-1 |  | + |  | <1 | 1 |  |  |  | + |  |  | 0 | 0 | + | + | + |
| **Glioblastoma multiforme IV** | | | | | | | | | | | | | | | | | | | | | |
| 16 | 22 |  | 20 | 2 | 1 |  | + |  | <1 | 1-2 |  |  |  |  |  |  | 0 | 0 | + | + | + |
| 17 | 50 |  | 20 | 2 | 1-2 |  |  |  | 8 | 1-2 |  |  |  |  |  |  | <1 | 1-2 | + |  | + |
| 18 | 61 |  | 30 | 1-2 | 0-2 |  |  |  | <1 | 1-2 |  |  |  |  |  |  | 1-2 | 1-2 | + | + | + |
| 19 | 74 |  | 8 | 1 | 1 |  |  |  | 0 | 0 |  |  |  |  |  |  | <1 | 1-2 | + |  | + |
| 20 | 49 |  | 0 | 0 | 0-2 |  |  |  | 0 | 0 |  |  |  | + |  |  | <1 | 1-2 | + | + | + |
| 21 | 75 |  | 3 | 1 | 0-1 |  |  |  | 0 | 0 |  |  |  |  |  |  | 7 | 2-3 |  |  |  |
| 22 | 63 |  | <1 | 1-2 | 0-1 |  | + |  | 0 | 0 |  |  |  | + | + |  | 0 | 0 |  | + |  |
| 23 | 68 |  | 0 | 0 | 0-1 | + |  |  | 0 | 0 |  |  |  | + |  |  | 0 | 0 | + | **+** | **+** |
| **Gliosarcoma IV** | | | | | | | | | | | | | | | | | | | | | |
| 24 | 41 |  | 40 | 2 | 1-2 |  |  |  | 0 | 1 |  | + |  |  |  |  | 0 | 0 | + |  | + |
| 25 | 53 |  | 70 | 1-2 | 0-1 |  |  |  | 1 | 1 |  |  |  |  |  |  | 0 | 0 | + | + | + |
| 26 | 69 |  | 15 | 1-2 | 0-1 |  | + |  | 0 | 0 |  | + |  |  |  |  | 1-2 | 1-2 | + | + | + |
| 27 | 66 |  | 0 | 0 | 0-2 |  |  |  | <1 | 1 |  |  |  | + |  |  | <1 | 1-2 |  |  | + |
| 28 | 46 |  | 0 | 1 | 0-1 |  |  |  | 0 | 0 |  |  |  |  |  |  | <1 | 1-2 | + |  | + |
| 29 | 57 |  | 0 | 0 | 0-1 |  | + |  | 0 | 0 |  |  |  |  |  |  | 0 | 0 |  |  | + |
| **Giant cell glioblastoma IV** | | | | | | | | | | | | | | | | | | | | | |
| 30 | 24 |  | 80 | 2 | 0-1 |  |  |  | <1 | 1-2 |  |  |  |  |  |  | 0 | 0 | + | + |  |
| 31 | 60 |  | 35 | 1-2 | 1 |  |  |  | <1 | 1 |  |  |  |  |  |  | 0 | 0 | + |  | + |
| 32 | 76 |  | 50 | 0-1 | 0-1 |  |  |  | 0 | 0 |  |  |  |  |  |  | 0 | 0 |  |  |  |
| 33 | 24 |  | 40 | 2 | 0-1 |  |  |  | 0 | 0 |  |  |  | + |  |  | 0 | 0 |  |  |  |
| **Oligodendroglioma II** | | | | | | | | | | | | | | | | | | | | | |
| 37 | 21 |  | 10 | 2 | 1-2 |  |  |  | 0 | 0 |  |  |  |  |  |  | 0 | 0 |  |  |  |
| 38 | 36 |  | 1-2 | 2 | 1-2 |  |  |  | 0 | 0 |  |  |  |  |  |  | 0 | 0 |  | + | + |
| 39 | 77 |  | 0 | 0 | 1-2 |  |  |  | 0 | 0 |  |  |  |  |  |  | 0 | 0 |  |  |  |
| 40 | 39 |  | 0 | 0 | 0-1 |  |  |  | 0 | 0 |  |  |  |  |  |  | 0 | 0 |  |  | + |
| 41 | 51 |  | 3 | 1-2 | 0-1 |  |  |  | 0 | 0 |  |  |  |  |  |  | 0 | 0 |  | + |  |
| 42 | 27 |  | <1 | 1 | 0-1 |  |  |  | 0 | 0 |  |  |  |  |  |  | 0 | 1 |  |  |  |
| 50 | 65 |  | <1 | 2 | 0-2 |  |  |  | <1 | 1 |  |  |  |  |  |  | 0 | 0 |  | + | + |
| 51 | 47 |  | <1-1 | 1-2 | 0 |  | + |  | 0 | 1-2 |  | + |  |  |  |  | 0 | 0 |  | + | + |
| 52 | 25 |  | <1 | 1-2 | 0-1 |  | + |  | <1 | 1-2 |  |  |  |  |  |  | <1 | 1-2 |  |  |  |
| **Anaplastic oligodendroglioma III** | | | | | | | | | | | | | | | | | | | | | |
| 43 | 32 |  | <1 | 1-2 | 0-2 |  |  |  | 0 | 0 |  |  |  |  |  |  | 0 | 0 |  |  | + |
| 44 | 36 |  | 30 | 1-2 | 0-1 |  |  |  | 1 | 1-2 |  | + |  |  |  |  | 0 | 0 | + |  |  |
| 45 | 38 |  | <1 | 3 | 0-1 |  |  |  | 0 | 0 |  | + |  |  |  |  | 0 | 0 |  | + |  |
| 46 | 37 |  | 0 | 0 | 0-1 |  |  |  | 0 | 0 |  |  |  |  |  |  | 0 | 0 |  | + |  |
| 47 | 32 |  | <1 | 1 | 0 |  |  |  | 0 | 0 |  | + |  |  |  |  | 0 | 0 | + |  |  |
| 57 (rec) | 41 |  | 0 | 2 | 0 |  |  |  | 0 | 0 |  |  |  |  |  |  | <1 | 1-2 |  | + | + |
| **Ganglioglioma I** | | | | | | | | | | | | | | | | | | | | | |
| 34 | 4 |  | <1 | 2-3 | 1-2 |  |  |  | 0 | 0 |  |  |  |  |  |  | 3 | 1-2 |  |  |  |
| 35 | 21 |  | 6 | 2 | 1-2 |  |  |  | <1 | 1 |  |  |  |  |  |  | <1 | 1 |  |  | + |
| 36 | 21 |  | 0 | 0 | 0 |  |  |  | <1 | 1 |  |  |  |  |  |  | 0 | 0 |  |  |  |
| **Oligoastrocytoma II (OA,NOS)** | | | | | | | | | | | | | | | | | | | | | |
| 48 | 20 |  | 20 | 2 | 1-2 |  |  |  | <1 | 1 |  |  |  |  |  |  | 0 | 0 | + |  |  |
| **Anaplastic oligoastrocytoma III (OAA,NOS)** | | | | | | | | | | | | | | | | | | | | | |
| 54 | 48 |  | 90 | 2 | 1 |  | + |  | <1 | 1-2 |  | + |  |  | + |  | 0 | 0 |  | + | + |

Dens, Density; Int, Intensity; DS, diffuse staining; N, neutrophil granulocytes; M, glioma-associated macrophages and microglia; BV, blood vessels; rec, recurrence.

**SUPPLEMENTARY TABLE 4|** Sequences of the forward and reverse primers (Microsynth, Balgach, Switzerland) used in RT-PCR analysis.

| **Gene** | **Fwd [5´ - 3´]** | **Rev [5´ - 3´]** |
| --- | --- | --- |
| RPL27 | GCTGGAATTGACCGCTACC | TCTCTGAAGACATTCTTATTGACG |
| GAL | CTGCTCGCCTCCCTCCTC | TGTCGCTGAATGACCTGTG |
| GAL_1_-R | TCTGCTTCTGCTATGCCAAGG | AGAGATGGATGATGTGGTGCG |
| GAL_2_-R | GCCGACCTGTGTTTCATCC | GGAGTGCAGCGGGTAG |
| GAL_3_-R | TTACGCTGGCTGCTGTCTCC | CGGTGCCGTAGTAGCTGAGGTA |

**SUPPLEMENTARY TABLE 5|** Overview of previous published data on GAL expression in different pituitary adenomas.

|  |  |  | **Cushing’s disease (ACTH)** | |  | **Acromegaly (GH)** | |  | **Prolactinoma** | |  | **Non-functioning tumor** | |
| --- | --- | --- | --- | --- | --- | --- | --- | --- | --- | --- | --- | --- | --- |
|  | **Total** |  | **n** | **GAL** |  | **n** | **GAL** |  | **n** | **GAL** |  | **n** | **GAL** |
| (Bennet et al., 1991) | 25 |  | 0 | 0 |  | 4 | 2 |  | 1 | 0 |  | 13 | 3 |
| (Hsu et al., 1991) | 62 |  | 19 | 16 |  | 11 | 5 |  | 14 | 2 |  | 18 | 9 |
| (Polak et al., 1990) | 23 |  | 6 | 6 |  | 8 | 3 |  | 2 | 2 |  | 7 | 0 |
| (Sano et al., 1991) | 42 |  | 14 | 9 |  | 10 | 0 |  | 9 | 0 |  | 9 | 0 |
| (Vrontakis et al., 1990) | 37 |  | 18 | 13 |  | 4 | 0 |  | 8 | 0 |  | 7 | 0 |
| (Leung et al., 2002) | 151 |  | 16 | 10 |  | 26 | 1 |  | 19 | 1 |  | 89 | 25 |
| (Grenback et al., 2004) | 24 |  | 7 | 7 |  | 6 | 5 |  | 1 | 1 |  | 10 | 9 |
| (Tofighi et al., 2012) | 13 |  | 2 | 2 |  | 2 | 2 |  | 1 | 1 |  | 8 | 8 |
| **Total** | **377** |  | **82** | **63**  **(77%)** |  | **71** | **18**  **(25%)** |  | **55** | **7**  **(13%)** |  | **161** | **54**  **(34%)** |

ACTH, adrenocorticotropic hormone; GH, growth hormone.

# References to Supplementary Table 5

Bennet, W.M., Hill, S.F., Ghatei, M.A., and Bloom, S.R. (1991). Galanin in the normal human pituitary and brain and in pituitary adenomas. *J Endocrinol* 130(3)**,** 463-467. doi: 10.1677/joe.0.1300463.

Grenback, E., Bjellerup, P., Wallerman, E., Lundblad, L., Anggard, A., Ericson, K., et al. (2004). Galanin in pituitary adenomas. *Regul Pept* 117(2)**,** 127-139. doi: 10.1016/j.regpep.2003.10.022.

Hsu, D.W., Hooi, S.C., Hedley-Whyte, E.T., Strauss, R.M., and Kaplan, L.M. (1991). Coexpression of galanin and adrenocorticotropic hormone in human pituitary and pituitary adenomas. *Am J Pathol* 138(4)**,** 897-909.

Leung, B., Iisma, T.P., Leung, K.C., Hort, Y.J., Turner, J., Sheehy, J.P., et al. (2002). Galanin in human pituitary adenomas: frequency and clinical significance. *Clin Endocrinol (Oxf)* 56(3)**,** 397-403. doi: 10.1046/j.1365-2265.2002.01486.x.

Polak, J.M., Gibson, S., Gentleman, S., Steel, J., and Van Noorden, S. (1990). *Galanin: distribution, ontogeny and expression following manipulation of the endocrine and nervous systems.* Macmillan Press, London. doi: 10.1007/978-1-349-12664-4_9.

Sano, T., Vrontakis, M.E., Kovacs, K., Asa, S.L., and Friesen, H.G. (1991). Galanin immunoreactivity in neuroendocrine tumors. *Arch Pathol Lab Med* 115(9)**,** 926-929.

Tofighi, R., Barde, S., Palkovits, M., Hoog, A., Hokfelt, T., Ceccatelli, S., et al. (2012). Galanin and its three receptors in human pituitary adenoma. *Neuropeptides* 46(5)**,** 195-201. doi: 10.1016/j.npep.2012.07.003.

Vrontakis, M.E., Sano, T., Kovacs, K., and Friesen, H.G. (1990). Presence of galanin-like immunoreactivity in nontumorous corticotrophs and corticotroph adenomas of the human pituitary. *J Clin Endocrinol Metab* 70(3)**,** 747-751. doi: 10.1210/jcem-70-3-747.
